# Classification of Task-State fMRI Data Based on Circle-EMD and Machine Learning

**DOI:** 10.1155/2020/7691294

**Published:** 2020-08-01

**Authors:** Renzhou Gui, Tongjie Chen, Han Nie

**Affiliations:** The Department of Information and Communication Engineering, Tongji University, Shanghai 201804, China

## Abstract

In the research work of the brain-computer interface and the function of human brain work, the state classification of multitask state fMRI data is a problem. The fMRI signal of the human brain is a nonstationary signal with many noise effects and interference. Based on the commonly used nonstationary signal analysis method, Hilbert–Huang transform (HHT), we propose an improved circle-EMD algorithm to suppress the end effect. The algorithm can extract different intrinsic mode functions (IMFs), decompose the fMRI data to filter out low frequency and other redundant noise signals, and more accurately reflect the true characteristics of the original signal. For the filtered fMRI signal, we use three existing different machine learning methods: logistic regression (LR), support vector machine (SVM), and deep neural network (DNN) to achieve effective classification of different task states. The experiment compares the results of these machine learning methods and confirms that the deep neural network has the highest accuracy for task-state fMRI data classification and the effectiveness of the improved circle-EMD algorithm.

## 1. Introduction

The brain-computer interface (BCI) allows people to control the machine through signals generated by brain activity to implement communications between people and the environment and expand the ability of humans to control machines [1]. In recent years, functional magnetic resonance imaging (fMRI) has become an important means of studying the high-level cortical function of the human brain [2–5]. Based on blood-oxygen-level-dependent (BOLD) imaging, it is a tool that allows functional changes to be studied in vivo [6] and has been used to display the morphology and location of the cortical center. The process of data acquisition is noninvasive and nonradiative, and the image resolution of the data is very high and easy to combine with conventional MR anatomical images.

One major feature of the fMRI signal is that it does not satisfy linearity and stationarity and has both temporal and spatial distribution characteristics [7, 8]. In 1999, Hilbert–Huang transform (HHT) [9], which is a combination of empirical mode decomposition (EMD) and Hilbert transform (HT), is an adaptive time-frequency analysis method and very suitable for feature extraction and analysis of the nonlinear and nonstationary signals [10]. HHT has good temporal and spatial resolution [11–13] and does not require a priori function basis [14], so that the original signal can be smoothed and decomposed to different scales of fluctuations and trends step by step [15]. The application field is very extensive [1, 16, 17] and very conducive to biomedical signal extraction [18].

However, a tricky problem with applying the EMD method is that when the cubic spline function is used to calculate both ends of the data sequence, an end effect occurs, causing the result to be distorted. In response to this problem, Deng et al. [19] used neural networks to extend the various intrinsic mode function (IMF) components to achieve accurate EMD decomposition. Yang and Jia [20] optimized the EMD and HT processes through time series modeling and prediction methods and achieved efficient frequency domain decomposition on stationary and simple nonstationary time series. Cheng et al. [21], Peng et al. [16], and Yang et al. [22] used the SVM approach to solve the end effect in the EMD method. Wang and Gang [23] proposed a new data expansion method based on minimum similarity distance, which effectively suppresses the divergence at both ends of the signal during EMD decomposition, thus improving the frequency resolution. On the basis of cubic spline interpolation, Tai and Deng [24] proposed an improved EMD method based on multiobjective optimization, which achieved a good solution. From the above, we can see that there are many improved methods for nonstationary signal decomposition, but there is still a need to find a suitable fast and efficient solution for spatial signals.

After smoothing the fMRI signal and extracting features, machine state learning can be used to classify feature learning and reconstruction, and so on for the task-state fMRI data. In recent years, many classification methods have been used and have important significance for fMRI research, such as support vector machine (SVM) [25], neighbor algorithm (kNN) [26], Gaussian Bayesian (GNB), linear discriminant analysis (LDA) [27], logistic regression (LR) [28], and deep learning [29–31]. Compared with traditional machine learning methods, deep learning has strong learning ability and can make better use of data sets for feature extraction [32]. Nowadays, the use of deep learning to achieve in-depth analysis of fMRI data has a very high practical value. However, at present, the high-precision classification of multitask state fMRI data is still a problem.

There are many noise effects in the task-state data, and the accuracy is generally lower than that of the rest state data. Xin et al. [33] proposed a new classification algorithm for depression, called weighted discriminant dictionary learning (WDDL) of fMRI data in task state, with an accuracy rate of 79.31%. Ertugrul et al. [34] proposed a new framework to encode the local connectivity patterns of the brain. They classify the cognitive state of the Human Connectome Project (HCP) task fMRI dataset by training the SVM. When characterizing the pairwise correlation between pairs of bold responses in all regions, the classification accuracy was 77.49%.

In view of the above analysis, we use EMD to process the spatial frequency information of fMRI data at each time point and improve the effectiveness of machine learning for fMRI data by stripping out the spatial frequency data of different stages. Therefore, based on the fMRI spatial data, this paper proposed an effective circle-EMD method for decomposing spatial nonstationary signals for the research and classification of multitask state fMRI data and solved the end effect of the traditional EMD method. We deeply studied and compared the characteristics and contribution of each IMF of fMRI data, verified the effectiveness of this method by using a variety of machine learning algorithms, and finally achieved a higher precision classification of fMRI spatial data.

## 2. Materials and Methods

### 2.1. Basic Principles of the EMD Method

The purpose of EMD is to decompose the signal into the sum of the IMFs and obtain the components of the original signal with different frequencies. The main steps of EMD can be summarized as follows:

1. First, local extrema including the maximum and minimum values of the signal *x*(*t*) are obtained, and the upper envelope signals *e*_upp_(*t*) and the lower envelope signal *e*_low_(*t*) are, respectively, constructed by cubic spline interpolation according to these values. And then we get the average of the envelope: *m*(*t*)=[*e*_upp_(*t*)+*e*_low_(*t*)]/2.
2. We calculate the signal *h*_1_(*t*) which has high frequency by reducing the average from the signal: *h*_1_(*t*)=*x*(*t*) − *m*(*t*).
3. If the signal *h*_1_(*t*) satisfies the two conditions of the IMF [35], (1) the function must have the same number of local extremum points and zero crossings over the entire time range, or at most one difference, and (2) at any point in time, the envelope of the local maximum (upper envelope) and the envelope of the local minimum (lower envelope) must be zero on average. Then, we set this signal to IMF and name it *C*_1_(*t*), and the residual signal *r*_1_(*t*)=*x*(*t*) − *C*_1_(*t*).
4. If the condition is not satisfied, the signals *h*_1_(*t*) are repeated as steps 1 and 2 as the original signal *x*(*t*), and it is worthwhile to obtain an IMF that satisfies the condition.
5. Steps 1∼3 are repeated until the residual signal *r*_*n*_(*t*) is a monotonic function or constant.

Finally, we decompose the signal to generate *n* IMFs and a residual signal, namely, *x*(*t*)=∑_*i*=1_^*n*^*C*_*i*_(*t*)+*r*_*n*_(*t*).

### 2.2. Improvement of EMD Algorithm: Circle-EMD

**Figure 1:**
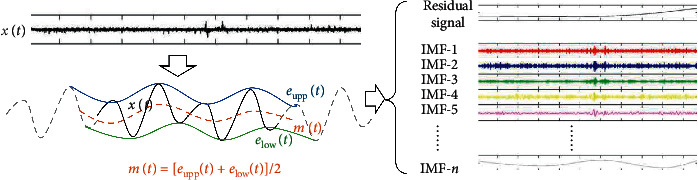
flowchart of the proposed circle-EMD.

Since the EMD algorithm uses a cubic spline difference to fit the envelope of the maxima and minima, it produces end effects at both ends. By analyzing the spatial signal sequence of fMRI (at one time), we find that the signals of each ROI of fMRI have no temporal correlations but have spatial correlation such as a net and should not be regarded as time series but network. Therefore, in this paper, the data queues of each ROI are connected to form a circle (as shown in the left part of Figure 1, the gray dotted lines are the back-end part and front-end part of the data supplementary to the front-end and back-end of this original data), so that there is no end effect when calculating the envelope, and it can better help extract the space feature.

Let the signal *x*(*t*) be *n* + 1 values from 0 to *n*, and *S*(*j*) denotes the fitting function between the *j*th and *j* + 1th values, and the second derivative of each endpoint is *S*_*j*_^″^=*M*_*j*_, (*j* = 0, 1, 2,…, *n*). Then, the traditional cubic spline interpolation equations are shown in the following equation:

(1) $$\begin{array}{c} \left\{\begin{array}{c} \mu_{1}M_{\text{0}}+2M_{1}+\lambda_{1}M_{2}=6f\left[t_{\text{0}},t_{1},t_{2}\right],\\ \mu_{2}M_{1}+2M_{2}+\lambda_{2}M_{3}=6f\left[t_{1},t_{2},t_{3}\right],\\ \ldots \\ \mu_{n-1}M_{n-2}+2M_{n-1}+\lambda_{n-1}M_{n}=6f\left[t_{n-2},t_{n-1},t_{n}\right]. \end{array}\right. \end{array}$$

There is a total of *n* − 1 equations and *n* + 1 unknown variables, where *h*_*j*_=*t*_*j*+1_ − *t*_*j*_=1, *μ*_*j*_=(*h*_*j*−1_/(*h*_*j*−1_+*h*_*j*_))=0.5, *λ*_*J*_=(*h*_*j*_/(*h*_*j*−1_+*h*_*j*_))=0.5, and *f*[*t*_*j*−1_, *t*_*j*_, *t*_*j*+1_]=(1/(*h*_*j*−1_+*h*_*j*_))[((*x*(*t*_*j*_) − *x*(*t*_*j*−1_))/*h*_*j*−1_) − ((*x*(*t*_*j*+1_) − *x*(*t*_*j*_))/*h*_*j*_)]=2*x*(*t*_*j*_) − *x*(*t*_*j*−1_) − *x*(*t*_*j*+1_). After connecting the beginning and end of the data queue, we set the fit function between *x*(*n*) and *x*(0) to *S*(*n*).

Through the formulas *S*^″^(*n*)_0_=*S*^″^(0)_*n*_ and *S*^″^(*n*)_*n*_=*S*^″^(*n* − 1)_*n*_, there are complementary equations:

(2) $$\begin{array}{c} \left\{\begin{array}{c} \mu_{0}M_{n}+2M_{0}+\lambda_{0}M_{1}=6f\left[t_{n},t_{0},t_{1}\right],\\ \mu_{n}M_{n-1}+2M_{n}+\lambda_{n}M_{0}=6f\left[t_{n-1},t_{n},t_{0}\right]. \end{array}\right. \end{array}$$

**Figure 2:**
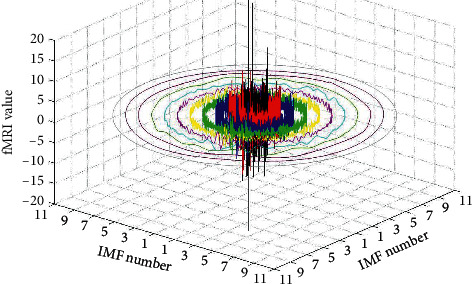
IMF signals with different frequency domains decomposed by circle-EMD.

At this time, a total of *n* + 1 equations and *n* + 1 unknown variables can calculate all the unknown coefficients. The individual IMF components of the EMD decomposition can be further determined by the new annular envelope, as shown in the right part of Figure 1. All the IMF signals decomposed by circle-EMD can also be regarded as a circular data, as shown in Figure 2, in which the black signal is the original signal and the other signals with other colors are IMF signals.

### 2.3. Machine Learning Algorithms and Models

We use three existing machine learning methods, LR, SVM, and DNN, to achieve effective classification of different task states.

#### 2.3.1. Nonlinear SVM

SVM is a binary classifier based on supervised learning and has been widely used in various scientific fields [36–38]. The boundary of classification is the maximum margin hyperplane for solving learning samples. When encountering some linearly inseparable problems (feature surfaces have hypersurfaces), nonlinear functions can be used to transform these into linear separable problems by mapping data from the original feature space to higher-dimensional Hilbert spaces. Common kernel functions in SVM are polynomial kernel, RBF kernel, Laplacian kernel, and Sigmoid kernel. This paper uses the RBF kernel function, also known as the Gaussian kernel, whose corresponding mapping function can map the sample space to an infinite dimensional space. The analytical expression is shown in the following equation:

(3) $$\begin{array}{c} K\left(X_{1},\;X_{2}\right)=\text{exp}\left(-\left(\left(X_{1}-X_{2}^{2}\right)|/|2\sigma^{2}\right)\right). \end{array}$$

#### 2.3.2. Logistic Regression for Multiclassification

LR is a generalized linear regression analysis model that can handle both classification and regression problems. The improved LR can even handle multiclassification problems [39]. The relationship between the intermediate value *y* and the input *x* represents the linear part of the model, which is *y*= ∑_*i*=1_^*m*^(*w*_*i*_∗*x*_*i*_)+*b*, where *w*_*i*_ is the weight matrix and *b* is the bias. Through the obtained *y*, the final result of the regression function can be obtained after passing the Softmax function. The formula for Softmax is *S*(*y*)=(1/(1+*e*^−*y*^)), whose the value range is distributed in [0,1].

#### 2.3.3. Deep Neural Network

The neural network was first developed into an important part [40] of machine learning by being inspired by neurons in the brain. The basis of neural networks is the perceptron model [41]. A deep neural network is a neural network that contains multiple layers of hidden layers. DNN can be divided into three categories according to the location of different layers: input layer, hidden layer, and output layer.

**Figure 3:**
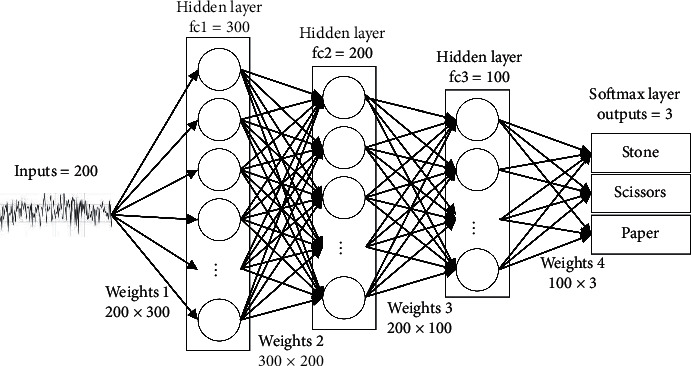
Deep learning model in this paper.

The deep learning model designed in this paper is shown in Figure 3. There are three hidden layers. The number of neurons in each layer is 300, 200, and 100, respectively. The activation function is ReLU function. Each layer uses Dropout and BatchNorm2d regularization to help prevent overfitting of the training. The loss function is the cross-entropy loss function, and the batch_size size is 32.

(4) $$\begin{array}{c} C=-\frac{1}{n}\sum_{x}\left[y\ln a+\left(1-y\right)\text{ln}\left(1-z\right)\right]. \end{array}$$

The cross-entropy represents the distance between the actual output (probability) and the expected output (probability), that is, the smaller the value of the cross-entropy, the closer the two probability distributions are. It is very helpful for the training of machine learning [42]. Assuming that the probability distribution *y* is the desired output, the probability distribution *z* is the actual output. The formula for the cross-entropy loss function is shown in the following equation:

We used 10-fold cross-validation to evaluate the model results. The data are divided into 10 groups, and 9 experiments are used as the training set and 1 group is used as the test set for each experiment. A total of 10 experiments were performed, so each group of data could be calculated as a test set. Finally, the accuracy of all test sets was averaged to obtain the final experimental results.

## 3. Experimental Process and Results

**Figure 4:**
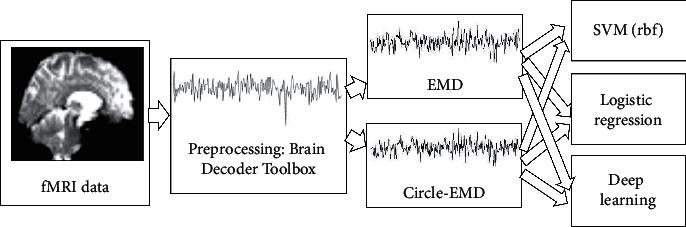
The whole process in this paper.

The experimental process is shown in Figure 4. fMRI data are first preprocessed by the Brain Decoder Toolbox, and then IMF signals are obtained by circle-EMD decomposition. Three different machine learning tools are used as experiments to verify the effective role of circle-EMD.

### 3.1. fMRI Data and Preprocessing

The data and fMRI decoding tools used in this article were obtained from the website (http://www.cns.atr.jp/dni/en/downloads/brain-decoder-toolbox/). The data in this paper were obtained on a 1.5 T MRI scanner (Shimadzu-Marconi) with a TR/TE/flip angle = 5 s/50 ms/90 degree, field of view (FOV) = 192 mm, acquisition matrix = 64 × 64, for functional images, and 50 slices. During the fMRI data acquisition process, the subjects performed three gestures of stone, scissors, and paper in the MRI according to the instructions. There are totally 10 runs, and each run has a 20-second break at the beginning and end. In each run, there are 32 states, including 8 rest states and 24 task states, so the task-state data have 240 samples. We divided into 10-fold cross-validation, 216 samples as the training set, and 24 samples as the test set per experiment, as shown in Table 1.

We use the Brain DecoderToolbox tool (the process flow and parameters are shown in Table 2) to smooth, decontour, and regularize the fMRI data and filter out the data of the six ROIs most relevant to the pose, which are “M1_RHand,” “SMA_RHand,” “CB_RHand,” “M1_LHand,” “SMA_LHand,” and “CB_LHand,” thus obtaining 5333 voxels for each sample. The locations of each ROI data are shown in Table 3. Based on the statistics of each voxel/channel (the data value is distributed between −5.44 and 24.12919), we filter out the top 200 data with the highest statistical value (the data value is distributed between 5.99434 and 24.12919) to initially filter unnecessary redundancy. (Through the actual classification and comparison in the experiment, if the original 5333 data are directly processed and classified without the filter, the accuracy of the classification is relatively low whether the SVM, LR, or DNN method is used).

By counting the position and number of six ROIs in the fMRI data sequence, we obtain the results shown in Table 3.

### 3.2. Decomposing Data by Using Circle-EMD

#### 3.2.1. Decomposed IMF Signal

**Figure 5:**
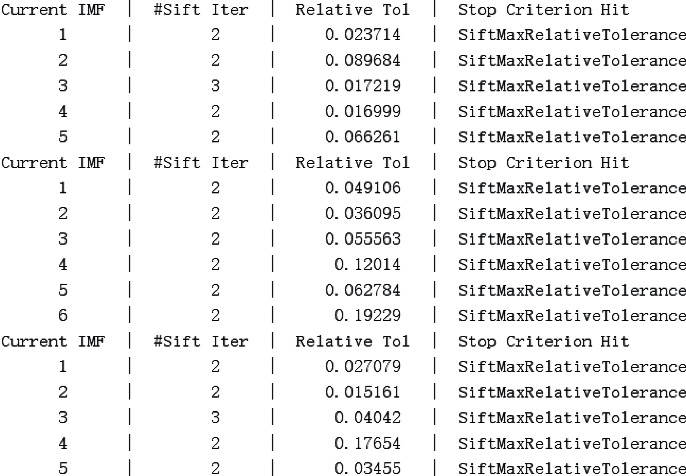
Circle-EMD results of decomposing fMRI signals.

As shown in Figure 5, it is a partial result of the circle-EMD decomposition of the preprocessed fMRI data sequence, where the “Current IMF” is the sequence number of the decomposed IMF signal and the “Sift Iter” is the number of iterations for extracting the IMF signal. For our data set, the number of IMFs decomposed is roughly between 4 and 7.

**Figure 6:**
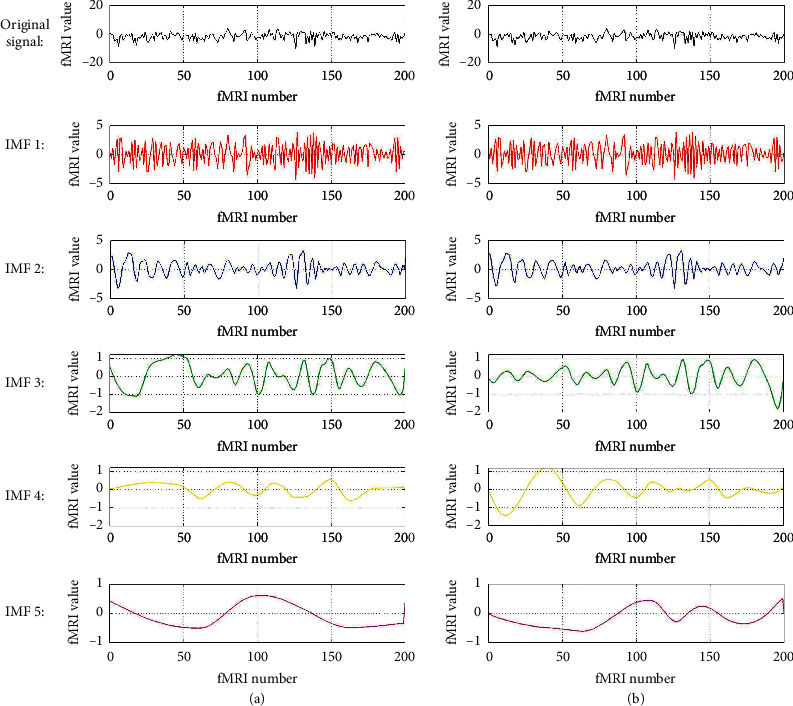
Each IMF signal. (a) Original EMD; (b) circle-EMD.

Using the original EMD and the circle-EMD algorithm proposed in this paper, we decompose the preprocessed fMRI data for each IMF signal, as shown in Figure 6. The uppermost black signal is an original data signal with a label of stone, and each IMF signal is decomposed by two EMD methods (the IMF signals are sequentially decomposed from top to bottom). By comparing the distribution results in Table 1, we find that the IMF signals obtained by the two decompositions are very similar in the high-frequency part. For example, in “CB_RHand,” these data have strong characteristic signals at both high and low frequencies. However, in the low-frequency part, the signal characteristics are more obvious, such as the data in “M1_RHand.”

**Figure 7:**
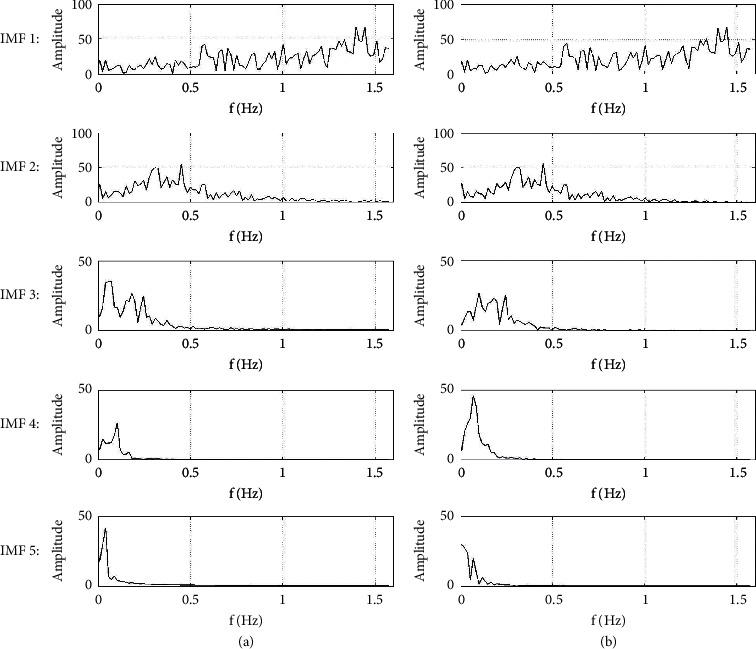
FFT frequency of each IMF signal. (a) Original EMD; (b) circle-EMD.

The frequency ranges for each IMF in Figure 6 are shown in Figure 7. For the convenience of display, fast Fourier transform (FFT) is used to transform these spatial IMF signals, and the frequency ranges are shown in the range of [−*π*/2, *π*/2] (only positive frequency is plotted). From Figure 7, it can also prove that the original signal has successfully been decomposed into IMF signals with different frequency bands and energy.

By further comparing more results between the two different methods, the common EMD algorithm has heavy end effects. The difference between the first and last of the signal is often interrupted. This means that when cubic spline interpolation is performed, there is a phenomenon of spectral leakage distortion after complementing 0, and the side lobes are large. This kind of signal has serious interference to the subsequent machine learning classification, which affects the classification accuracy.

**Figure 8:**
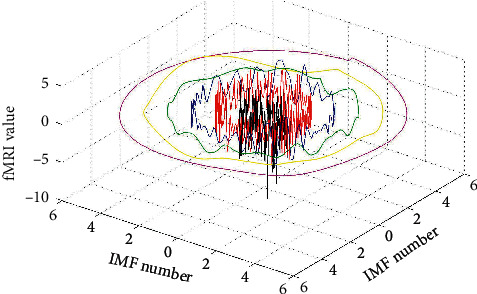
Original signal and each decomposed circle-IMF signal.

The data signal in this paper should not have a temporal correlation, nor the order of the data. The IMF signal decomposed by our algorithm has better continuity at the first and last of the signal, as shown in Figure 8. Thus, the signal does not have a starting position and an ending position in the actual sense and can be connected in a circle shape. And it is truly in the low-frequency signal portion, the signals have more significant data characteristics. Since there is no zero-compensation operation, there is no spectral distortion and the side lobes are extremely low, which can help improve the accuracy of classification.

#### 3.2.2. The Role of Different IMFs

We use the SVM method with RBF kernal to classify each IMF signal and signals formed by IMF combination, and view the contribution of each IMF signal to the classification. SVM uses MATLAB's libsvm-mat tool, version 3.0-1 (https://www.csie.ntu.edu.tw/∼cjlin/libsvm/), the kernel function parameter is set to degree = 3, gamma = 0, Coef0 = 0, nu = 0.5, cache memory size = 100, and tolerance of termination criterion = 1*e* − 3, *p*=0.1, shrinking = 1, and the weight matrix after training is 200 ∗ 3.

The average accuracy of the 10-fold cross-validation results is statistical as shown in Table 4. Since the number of IMFs obtained by decomposing each fMRI signal is not fixed, here we only extract the first 4 IMF signals when comparing the IMF separately and finally show the results of all IMF accumulations. In the right half of the table, we have realistic SVM predictions and real label statistics. For example, the third value of 40 in the first row indicates that 40 samples were correctly predicted under the 80 samples with the real label as stones.

By comparison, we find that the IMF signal decomposed first has higher frequency characteristics and also has a higher contribution to the classification result. The more the IMF signals are superimposed, the higher the accuracy of the data. Comparing with Table 5, we found that although the classification accuracy of a single IMF signal, there is no high accuracy of the original signal. However, after superimposing each IMF signal, that is, deleting the residual monotonic signal, the classification accuracy is the highest.

### 3.3. Classification Results of Machine Learning

**Figure 9:**
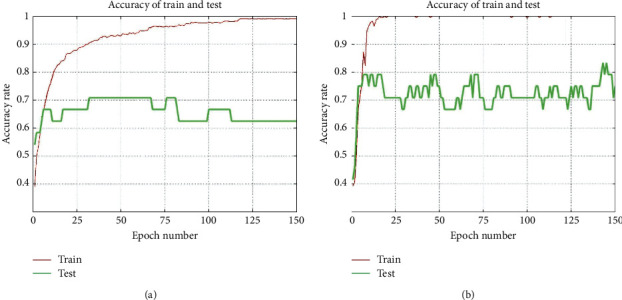
Accuracy rate curve. (a) Logistic regression. (b) Deep neural networks.

There are 240 data sets in this paper. In each experiment, 216 are training data and 24 are test sets. For each training data, the input is 200-dimensional fMRI data, and the output label is a 3-dimensional classified data. Using LR, SVM, and DNN, we classify raw data and data after preprocessed by circle-EMD. In the training process, the accuracy curve of the logistic regression and deep learning of the experiment is shown in the Figure 9 (red lines indicate the training set and green lines indicate the test set), and the accuracy rate classification results are shown in Table 6. To further compare the original signal with our circle-EMD method, we still do the machine learning classification statistics for the original signal.

From Table 6, we can see that for the fMRI data of this paper, no matter which machine learning method, our circle-EMD algorithm has achieved higher accuracy, which proves that our circle-EMD method is effective for separating spatial signals. Sex can help to refer to the classification accuracy of machine learning.

To further compare the sensitivity and specificity of the classification, this paper further calculated the weighted average of precision, recall, and f1-score of the classification results in Figure 6 (we only show the average value of 10-fold cross-validation results. And since the samples are balanced, the results of macroaverage and weighted average are the same for multiclassification mean calculation, so this paper only shows the results of weighted average).

Comparing the results of each machine learning classification, deep learning performed best in each experiment, and the average accuracy of the final three categories of the test set reached 81.2%. LR and DNN have achieved close to 100% classification accuracy on the training set of this data, but the performance of LR on the test set is not enough, and it does not have good generalization. This result not only confirms the validity of our circle-EMD method, but also the importance of deep learning for fMRI data classification and learning.

## 4. Conclusions

In this paper, we propose a circle-EMD algorithm to decompose and preprocess the “stone-scissors-paper” multitask state fMRI data and compare it with the original EMD method. On this basis, the three commonly used machine learning methods: logistic regression, SVM, and DNN are used to process the data obtained by different EMD algorithms. We compare the effects of different EMD algorithms on the processing results through experiments and finally obtain the classification accuracy of different data by different machine learning methods.

The results show that the proposed algorithm can eliminate the end effect of spatial signals and extract signals with the different spatial frequency features of multitask state data more effectively. It also reduces the effects of low frequencies and noise, more accurately reflects the data characteristics of the original signal (especially, the characteristics of the relatively low-frequency part), shows the correlation between different ROIs, and finally achieves higher classification accuracy. By comparing the various IMF signals extracted by this method, it is found that the contribution from low frequency to high frequency increases in turn, and the accumulation of multiple IMF signals helps to improve the accuracy of classification. Through comparison with different machine learning algorithms, we also found that the most effective algorithm is the deep learning algorithm. The highest accuracy in this experiment is up to 87.5%, with an average of 81.2%.

In general, the method of this paper provides a new circle-EMD-based data analysis method for analyzing task-state fMRI data and combines the deep learning method to provide a more effective solution for classification of multitask state fMRI data, helping to better realize the brain-computer interaction. The limitation of the combination of the algorithm and machine learning is that when processing spatial fMRI signals with small amount of data, the effect is more obvious (for example, 200 voxels are selected in this paper), and the use of decomposed IMF signals is only verified in the motion data type of this paper. In future research work, we will experiment with more types of fMRI data and try to weight each IMF signal to get a better performance and pay more attention to the residual signal when using circle-EMD.

## Figures and Tables

**Table 1:** Number of samples per experiment.

Data type	Rock	Scissors	Paper
Training data	72	72	72
Testing data	8	8	8

**Table 2:** Process name and parameters used when using the Brain Decoder Toolbox.

Order index of process flow	Process name	Parameters
1	shiftData	shift = 1 (shiftTR)
2	fmri_selectRoi	rois_use = {1, 1, 1, 1, 1, 1}
3	selectChanByTvals	num_chans = 200(nVoxels); tvals_min = 3.2
4	reduceOutliers	std_thres = 4; num_its = 2
5	detrend_bdtb	None
6	normByBaseline	base_conds = 1 : 4
7	zNorm_bdtb	app_dim = 2(alongspace); smode = 1
8	selectConds	conds = 2 : 4

**Table 3:** The locations of each ROI datum in the fMRI data sequence.

ROI name	TR/TE/flip angle	SMA_RHand	CB_RHand	M1_LHand	SMA_LHand	CB_LHand
Index	5 s/50 ms/90 degree	75-76, 85–88, 93, 95–109	110–114, 116–151	115, 152–200	75-76, 85–88, 93, 95–109	67, 71–74, 77–84, 89–92, 94

**Table 4:** Classification results of the task states of each circle-IMF signal and combined signals by using SVM.

IMF	Label	Rock (80)			Scissors (80)			Paper (80)		
	Prediction	Rock	Scissors	Paper	Rock	Scissors	Paper	Rock	Scissors	Paper
1	0.5625	40	20	20	18	43	19	10	18	52
2	0.4833	46	25	9	31	26	23	14	22	44
3	0.4292	47	12	21	33	20	27	23	21	36
4	0.4458	51	19	10	32	30	18	25	29	26
1 + 2	0.5875	44	27	9	24	40	16	7	16	57
1 + 3	0.60	46	17	17	18	44	18	10	16	54
1 + 4	0.575	41	23	16	17	46	17	11	18	51
1 + 2 + 3	0.6333	50	20	10	22	45	13	7	16	57
1 + 2 + 4	0.5958	48	22	10	18	43	19	8	20	52
1 + 2 + 3 + 4	0.6417	53	18	9	20	45	15	6	18	56
All IMFs	0.6542	53	18	9	19	47	14	7	16	57

**Table 5:** Classification results of original signals that have not been processed by EMD.

	Label	Rock (80)			Scissors (80)			Paper (80)		
	Prediction	Rock	Scissors	Paper	Rock	Scissors	Paper	Rock	Scissors	Paper
Original signal	0.6375	50	21	9	21	45	14	6	16	58

**Table 6:** Accuracy results of extracted fMRI signals by using different machine learning algorithms.

Experiment number	Logistic regression		SVM		Deep learning	
	Original	All IMFs	Original	All IMFs	Original	All IMFs
1	0.667	0.750	0.708	0.708	0.833	**0.875**
2	0.667	0.708	0.667	0.708	0.792	**0.875**
3	0.625	0.667	0.708	0.708	0.708	**0.833**
4	0.667	0.708	0.583	0.625	0.667	**0.792**
5	0.667	0.625	0.583	0.542	0.750	0.750
6	0.708	0.708	0.625	0.667	0.750	**0.792**
7	0.708	0.667	0.583	0.583	0.792	**0.833**
8	0.667	0.667	0.542	0.583	0.542	**0.708**
9	0.708	0.750	0.625	0.667	0.750	**0.833**
10	0.750	0.750	0.750	0.750	0.750	**0.833**
Average	0.683	0.700	0.638	0.654	0.733	**0.812**

**Table 7:** Sensitivity and specificity of the classification.

Weighted average	Logistic regression		SVM		Deep learning	
	Original	All IMFs	Original	All IMFs	Original	All IMFs
Precision	0.690	0.711	0.468	0.486	0.745	**0.825**
Recall	0.683	0.700	0.638	0.654	0.733	**0.812**
f1-score	0.681	0.696	0.538	0.557	0.729	**0.806**

## Data Availability

For original fMRI data address, please visit https://www.nitrc.org/projects/bdtb/. The processed data used to support the findings of this study are available from the corresponding author upon request.
